# Construction and validation of an autophagy‐related long noncoding RNA signature for prognosis prediction in kidney renal clear cell carcinoma patients

**DOI:** 10.1002/cam4.3820

**Published:** 2021-03-02

**Authors:** JunJie Yu, WeiPu Mao, Bin Xu, Ming Chen

**Affiliations:** ^1^ Department of medical college Southeast University Nanjing China; ^2^ Department of Urology Southeast University Zhongda hospital Nanjing China

**Keywords:** autophagy, kidney renal clear cell carcinoma, long noncoding RNA, prognostic signature, The Cancer Genome Atlas

## Abstract

**Purpose:**

The purpose of this study was to identify autophagy‐associated long noncoding RNAs (ARlncRNAs) using the kidney renal clear cell carcinoma (KIRC) patient data from The Cancer Genome Atlas (TCGA) database and to construct a prognostic risk‐related ARlncRNAs signature to accurately predict the prognosis of KIRC patients.

**Methods:**

The KIRC patient data were originated from TCGA database and were classified into a training set and testing set. Seven prognostic risk‐related ARlncRNAs, identified using univariate, lasso, and multivariate Cox regression analysis, were used to construct prognostic risk‐related signatures. Kaplan–Meier curves and receiver operating characteristic (ROC) curves as well as independent prognostic factor analysis and correlation analysis with clinical characteristics were utilized to evaluate and verify the specificity and sensitivity of the signature in training set and testing set, respectively. Two nomograms were established to predict the probable 1‐, 3‐, and 5‐year survival of the KIRC patients. In addition, the lncRNA‐mRNA co‐expression network was constructed and Gene Ontology (GO) enrichment analysis and Kyoto Encyclopedia of Genes and Genomes (KEGG) were used to identify biological functions of ARlncRNAs.

**Results:**

We constructed and verified a prognostic risk‐related ARlncRNAs signature in training set and testing set, respectively. We found the survival time of KIRC patients with low‐risk scores was significantly better than those with high‐risk scores in training set and testing set. ROC curves suggested that the area under the ROC (AUC) value for prognostic risk score signature was 0.81 in training set and 0.705 in testing set. And AUC values corresponding to 1‐, 3‐, and 5 years of OS were 0.809, 0.753, and 0.794 in training set and 0.698, 0.682, and 0.754 in testing set, respectively. We established the two nomograms that confirmed high C‐index and accomplished good prediction accuracy.

**Conclusions:**

We constructed a prognostic risk‐related ARlncRNAs signature that could accurately predict the prognosis of KIRC patients.

## 1 INTRODUCTION

Renal cell carcinoma was one of the most common solid malignant tumors of the urinary system, accounting for 3%–5% of all new tumors each year,^1^ of which KIRC accounts for 70%–80% of all renal cell carcinomas.^2^ Despite the emergence of many new targeted drugs and therapeutic strategies,^3^ the prevalence and mortality rates of KIRC have still increased annually. Moreover, KIRC was resistant to radiotherapy and chemotherapy,^4^ and surgery was the primary effective treatment method for localized KIRC, but had limited efficacy in advanced KIRC.^3^ It has been reported that about 30% of patients with KIRC has already developed metastases at first diagnosis.^5^ Therefore, to construct an effective prognostic prediction signature was of significant importance for the management of patients with KIRC.

Autophagy was a basic cellular metabolic process among all eukaryotic organisms in which damaged organelles and macromolecules were transported to the lysosome for degradation and recycling through various routes.^6^, ^7^ Autophagy had different roles in tumorigenesis, maintenance, and tumor progression.^8^ It had been reported that autophagy exerted a tumor‐suppressive role during tumor initiation and malignant transformation.^8^ On the contrary, it played a protective mechanism in cancer progression. Choi ME^9^ reported that autophagy had a therapeutic benefit in the treatment of RCC. Therefore, it was urgent to identify autophagy‐associated biomarkers for the early diagnosis and prognosis of patients with KIRC.

Long noncoding RNAs (lncRNAs) were defined as >200 nucleotides in length that could not encode proteins.^10^ Accumulating evidence^11^, ^12^, ^13^ suggested lncRNAs were involved in multiple biological processes, including tumor proliferation, differentiation, apoptosis, drug resistance, and metastasis, indicated that targeting lncRNAs might be a new approach for the diagnosis and treatment of patients with KIRC. It has also been reported that lncRNAs were involved in biological progression of KIRC by modulating autophagy.^14^

Therefore, we inferred that autophagy‐associated lncRNAs could be a valuable diagnostic and therapeutic indicator for KIRC patients. The aim of our study was to identify autophagy‐associated lncRNAs (ARlncRNAs) from The Cancer Genome Atlas (TCGA) database and constructed a prognostic‐related ARlncRNAs signature to predict the prognostic outcomes of the patients with KIRC accurately.

## 2 METHODS

### 2.1 Data source and preparation

Transcriptome profiling data in FPKM format and clinical data in XML format of 530 KIRC patients were downloaded from TCGA data portal (https://portal.gdc.cancer.gov/). Using Perl program, these FPKM data were collated and annotated, and then, sorted into protein‐coding genes and long noncoding RNAs by the Ensembl human genome browser (http://asia.ensembl.org/info/data/index.html).^15^ Clinical data were collated and excluded cases with survival times of less than 30 days (n = 17), to remove the possibility of non‐tumor death. A Total of 232 autophagy‐related genes (ARGs) were identified from the Human Autophagy Database (HADb) (http:// autophagy.lu/clustering/index.html). Pearson correlation analysis was adopted to evaluate the correlation between the expression of ARGs and lncRNAs. A correlation coefficient that met the criteria |R|^2^ > 0.8 and *p* < 0.001 was defined as an autophagy‐associated lncRNA (ARlncRNA). Since the data were extracted from TCGA database, following the publication guidelines strictly approved by TCGA, ethics committee approval was not required.

### 2.2 Construction of a prognostic risk‐related ARlncRNA signature model

To construct an effective prognostic prediction model, the ARlncRNAs expression matrix and clinical data were integrated. The samples were randomly classified into a training set (309 samples) and a testing set (204 samples) in a 3:2 ratio. We applied the training set to construct a prognostic signature and evaluated it in the testing set.

The univariate Cox proportional hazards regression analysis was applied to identify those ARlncRNAs significantly linked with prognosis (*p* < 0.01) in training set. Lasso regression analysis was used to eliminate those prognostic‐related ARlncRNAs positively correlated with each other to avoid overfitting. Later, the prognostic risk‐related ARlncRNAs were subjected to multivariate Cox proportional hazards regression analysis to determine independent prognostic factors. Ultimately, we constructed seven prognostic risk‐related ARlncRNAs as candidates for the prognostic signature. The risk score was used as a predictor of prognostic status in the model, calculated using the format $risk\;score\;=\;\sum_{i\;=\;1}^{n}coef\left(i\right)*\mathrm{lncRNA}\left(i\right)\;\mathrm{expression}$.

### 2.3 Evaluation of the prognostic signature in training group and verification in testing group

The KIRC patients were classified into high‐risk score group and low‐risk score group based on median risk score as the cutoff value in training set. The Kaplan–Meier survival curve was performed to compare the survival outcomes of the two groups. The receiver operating characteristic curves (ROC) were utilized to assess the specificity and sensitivity of the model as well as the accuracy at 1‐, 3‐, and 5 years by survival ROC and time‐survival ROC package.

We ranked KIRC patients according to the risk score. Then, the risk Score distribution and the number of censored patients, as well as prognosis‐related lncRNAs were visualized in high‐ and low‐risk group by distribution curves, scatter dot plot, and heatmap. We further evaluated the accuracy of the prognostic risk signature using the same methods in the testing set.

### 2.4 Independent prognostic factor analysis and correlation analysis with clinical characteristics

To assess the accuracy of the prognostic model in terms of prognostic survival outcomes, Cox regression analysis was utilized to validate independent risk factors. Multivariate ROC curves included traditional clinical variables and risk score further validated the predictive accuracy of the model.

### 2.5 Establishment and validation of prognostic nomograms

Two nomograms were established to predict the probable 1‐, 3‐, and 5‐year survival of the KIRC patients in training set, respectively. One integrated traditional clinical variable, including age, sex, AJCC stage, grade, TNM stage as well as risk score; the other integrated prognostic‐related ARlncRNAs and risk score. Afterward, the concordance index (C‐index) and calibration curves were used to evaluate the concordance between predicted survival outcomes and observed survival outcomes in training set and testing set.

### 2.6 Construction of a LncRNA‐mRNA co‐expression network and functional enrichment analysis

We constructed and visualized the lncRNA‐mRNA co‐expression network to assess the correlation between prognostic‐related ARlncRNAs and targeted mRNA using the Cytoscape software. Pearson correlation analysis was subjected to identify the targeted mRNAs connected to those ARlncRNAs based on correlation coefficient |R|^2^ > 0.3 and *p* < 0.05. Using the clusterProfiler package in R software, Gene Ontology (GO) enrichment analysis of targeted mRNA was utilized to identify molecular functions (MF), cellular components (CC), and biological processes (BP) associated with ARlncRNAs. The potential signaling pathways of the ARlncRNAs were elucidated using the Kyoto Encyclopedia of Genes and Genomes (KEGG).

### 2.7 Statistical analysis

The statistical analysis was performed in R software (version 4.1). The Perl programming language (Version 5.30.2) was used for data processing. *p* < 0.05 was regarded as statistically significant.

## 3 RESULTS

### 3.1 Construction of a prognostic risk‐related ARlncRNAs signature model in training set

**FIGURE 1:**
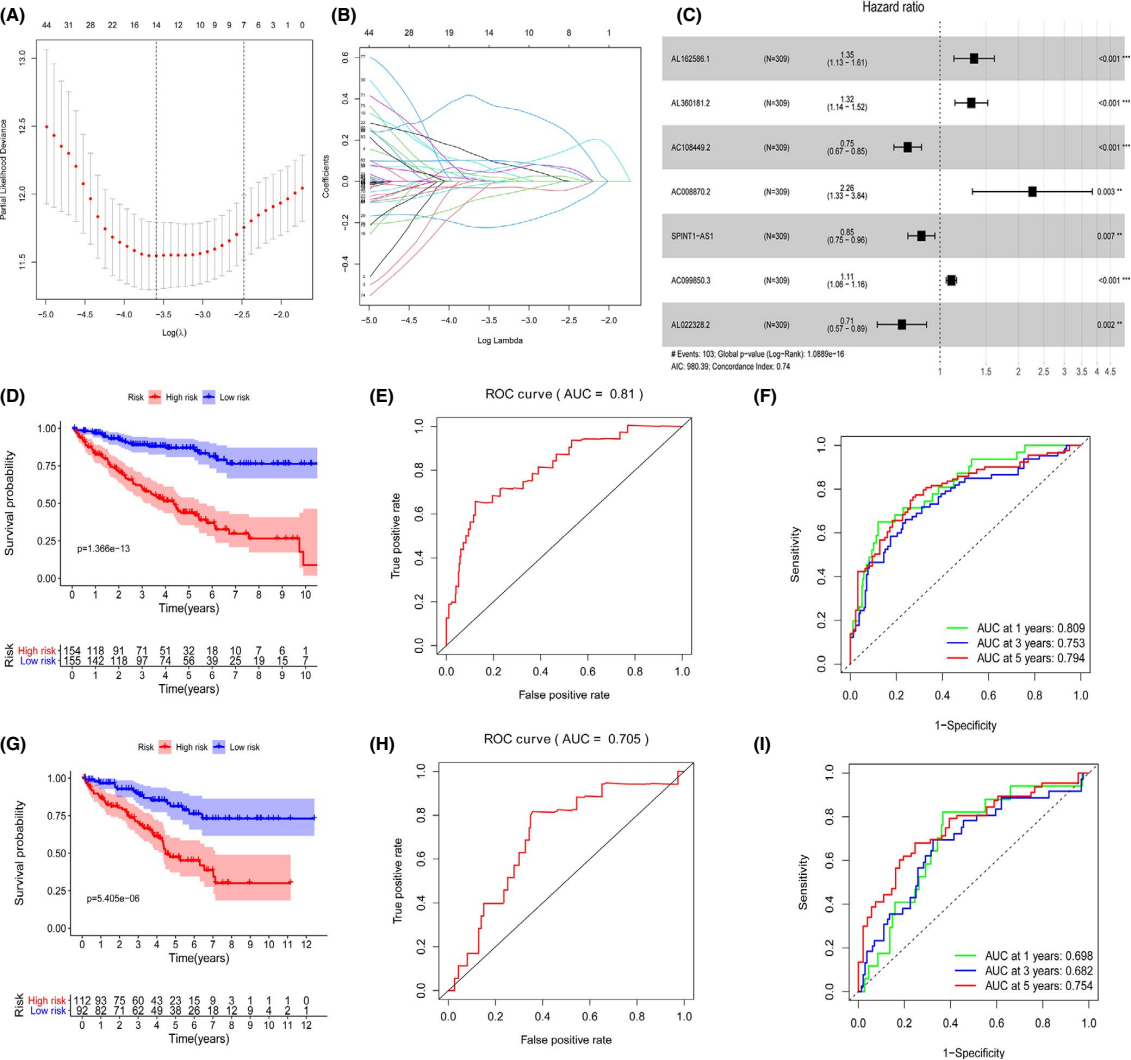
Construction and evaluation of prognostic‐related ARlncRNAs signature in training group and verification in testing group. Lasso regression analysis was performed to avoid overfitting in training group. (A) Lasso coefficient values and vertical dashed lines at the best log (lambda) value were displayed. (B) Lasso coefficient profiles of the prognostic lncRNAs. (C) Forest plot of multivariate cox regression analysis for seven prognostic‐related ARlncRNAs. The Hazard Ratio (HR) value and its 95% confidence interval with associated *p*‐value were showed. An HR of greater than 1 indicates that high gene expression was bad for the prognosis. These HRs greater than 1 were risk factors, which indicated that high expressions of lncRNAs were unfavorable for prognosis, while HRs less than 1 were protective factors, which indicated that high expressions of lncRNAs were favorable for prognosis. Kaplan–Meier survival curve for KIRC patients with high‐ and low‐risk scores in the training group (D) and testing group (G) ROC curves for the signature and its AUC value in training group (E) and testing group (H). ROC curves and their AUC value represented 1‐, 3‐, and 5‐year predictions in training group (F) and testing group (I)

**FIGURE 2:**
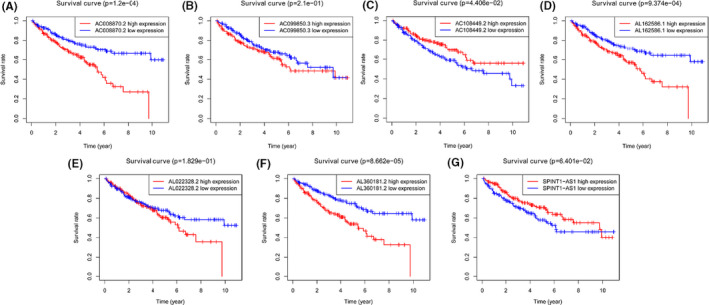
The Kaplan–Meier (KM) survival curve of seven prognostic‐related autophagy‐associated lncRNA (ARlncRNAs). (A) The KM survival curves for survival times of AC008870.2 in the high‐ and low‐risk group; (B) The KM survival curves for OS of AC099850.3 in the high‐ and low‐risk groups; (C) The KM survival curves for OS of AC108449.2 in the high‐ and low‐risk groups; (D) The KM survival curves for OS of AL162586.1 in the high‐ and low‐risk groups; (E) The KM survival curves for OS of AL022328.2 in the high‐ and low‐risk groups; (F) The KM survival curves for OS of AL360181.2 in the high‐ and low‐risk groups; (G) The KM survival curves for OS of SPINT1‐AS1 in the high‐ and low‐risk groups

A total of 14,142 lncRNAs were obtained by analyzing transcriptome profiling data of KIRC patients from TCGA database. We also identified 232 autophagy‐related genes from the HADb database. Then, Pearson correlation analysis between these lncRNAs and ARGs was performed and identified 1640 ARlncRNAs. Subsequently, we performed univariate Cox proportional hazards regression analysis of expression of the 1640 ARlncRNAs in the training set. We found that the expressions of 146 lncRNAs were significantly linked with prognosis of KIRC patients (*p* < 0.01). Lasso regression analysis and multivariate Cox proportional hazards regression analysis were adopted for the 146 prognostic‐related ARlncRNAs. Ultimately, as shown in Figure 1C, seven ARlncRNAs were identified to construct the prognostic risk score model using the formula as following: risk score = (0.30073387) * AL162586.1+(0.276333249) *AL360181.2+(−0.285774883) * AC108449.2+(0.816011076) * AC008870.2+(−0.164744933) *SPINT1‐AS1+(0.100233745) * AC099850.3+(−0.336573259) * AL022328.2. Furthermore, as shown in Table 1 and Figure 2, we found that AL162586.1, AL360181.2, AC008870.2, and AC099850.3 were risk factors for HR>1, whereas AC108449.2, SPINT1‐AS1, and AL022328.2 were favorable factors with HR<1.

**TABLE 1:** Multivariate Cox results of prognostic‐related ARlncRNAs based on TCGA data

lncRNA	coef	HR	HR.95%L	HR.95%H	*p*‐value
AL162586.1	0.300734	1.35085	1.130046	1.614797	0.000958
AL360181.2	0.276333	1.318287	1.140606	1.523647	0.000183
AC108449.2	−0.28577	0.751432	0.665034	0.849054	4.52E−06
AC008870.2	0.816011	2.261461	1.332053	3.839341	0.002514
SPINT1‐AS1	−0.16474	0.84811	0.752939	0.95531	0.006672
AC099850.3	0.100234	1.105429	1.055993	1.15718	1.76E−05
AL022328.2	−0.33657	0.714214	0.574523	0.887869	0.002437

### 3.2 Evaluation of the prognostic signature in training group and verification in testing group

To further validate the reliability of the prognostic risk‐related signature model, the Kaplan–Meier survival curves were performed in training set and testing set. It showed that the OS of KIRC patients with low‐risk score were significantly better than those with high‐risk score in training group (Figure 1D) and testing group (Figure 1G) (all *p* < 0.001). The 5‐year survival rates were 42.2% and 85.1% for the high‐risk and low‐risk score patients in training group and 45.0% and 78.8% in testing group. ROC curves showed that the area under the ROC (AUC) value for the prognostic risk‐related signature model was 0.81 in training group (Figure 1E) and 0.705 in testing group (Figure 1H). And AUC value corresponding to 1‐, 3‐, and 5 years of OS were 0.809, 0.753, and 0.794 in training set (Figure 1F) and 0.698, 0.682, and 0.754 in testing set (Figure 1I), respectively.

**FIGURE 3:**
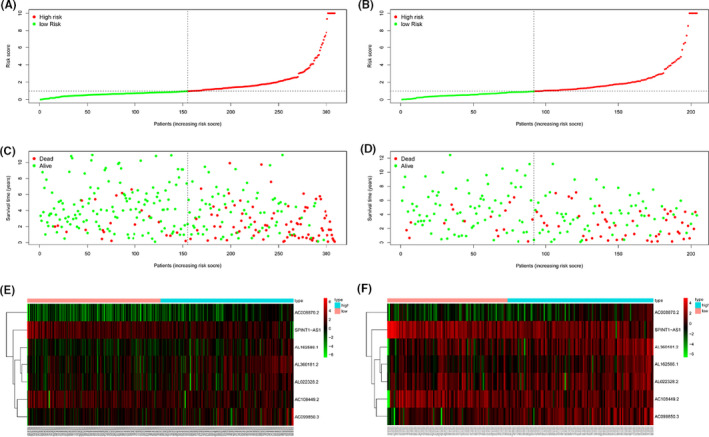
Evaluation of the prognostic signature in training group and verification in testing group. The risk Score distribution in high‐ and low‐risk score KIRC patients in training group (A) and testing group (B) scatter dot plot showed survival outcomes in high‐ and low‐risk KIRC patients in training group (C) and testing group (D). Heatmap showed the expressions of seven prognostic‐related autophagy‐associated lncRNAs (ARlncRNAs) in high‐ and low‐risk score KIRC patients in training group (E) and testing group (F)

The risk Score distribution was displayed in high‐ and low‐risk KIRC patients in training set (Figure 3A) and testing set (Figure 3B) and scatter dot plot showed that high‐risk score patients had worse survival outcomes than low‐risk score group based on prognostic‐related ARlncRNAs signature in training set (Figure 3C) and testing set (Figure 3D). The heatmap showed that high‐risk score KRIC patients expressed higher levels of risk factors, whereas low‐risk score KRIC patients expressed higher levels of protective factors, which suggested that there were significant differences between the seven prognostic‐related ARlncRNAs in high‐ and low‐risk score KIRC patients in training set (Figure 3E) and testing set (Figure 3F). AL162586.1, AL360181.2, AC008870.2, and AC099850.3 were risk factors that were upregulated in high‐risk score group, whereas AC108449.2, SPINT1‐AS1, and AL022328.2 were protective factors, which were downregulated in high‐risk score group.

### 3.3 Clinical value of the prognostic risk‐related ARlncRNA signature model

**FIGURE 4:**
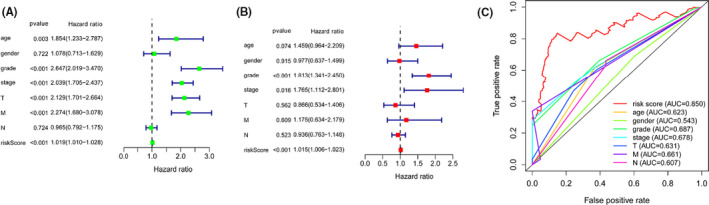
Estimation of clinical Value of the prognostic‐related ARlncRNA Signature and clinicopathological variables in KRIC patients. (A) The forest plots for univariate Cox regression analysis showed that risk score, age, grade, AJCC stage, T stage, and N stage were prognostic risk‐related variables. (B) The forest plots for multivariate Cox regression analysis showed risk score, grade, and AJCC stage were independent prognostic factors. (C) Multivariate receiver operating characteristic (ROC) curve analysis showed predictive accuracy of the model: the AUC value of risk score was higher than other clinicopathological variables

**FIGURE 5:**
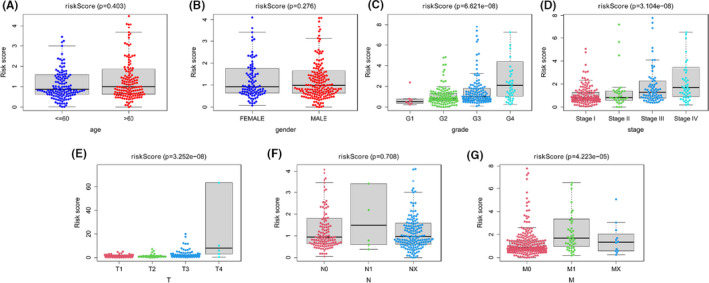
The correlation analysis of the risk score from prognostic signature with clinicopathological characteristics in the KIRC patients. The correlation between risk score and clinicopathological characteristics stratified according to (A) age (< =60 years, n = 258 vs. >60 years, n = 249); (B) gender (FEMALE, n = 175 vs. MALE, n = 332); (C) tumor grades (G1 grade, n = 12 vs. G2 grade, n = 219 vs. G3 grade, n = 201vs. G4 grade, n = 75); (D) AJCC stages (stages I, n = 253 vs. stages II, n = 53 vs. stages III, n = 118 vs. stages IV, n = 83); (E) T stage (T1, n = 259 vs. T2, n = 65 vs. T3, n = 172 vs. T4, n = 11); (F) N stage (N0, n = 226 vs. N1, n = 15 vs. Nx, n = 266); (G) M stage (M0, n = 405 vs. M1, n = 78 vs. Mx, n = 24)

**FIGURE 6:**
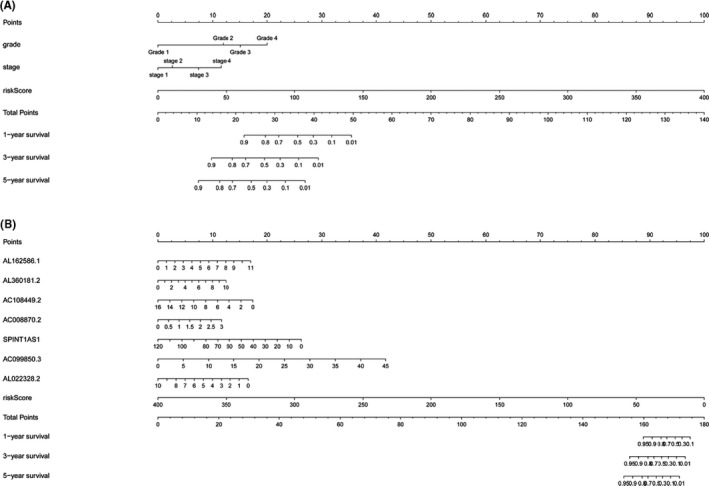
(A) Construction of a prognostic nomogram utilized risk score from the prognostic‐related ARlncRNAs signature and clinicopathological parameters clarified from multivariable Cox regression analysis to predict 1‐, 3‐, and 5‐year survival rate of KIRC patients. (B) Construction of a prognostic nomogram utilized risk score from the prognostic‐related ARlncRNAs signature and seven ARlncRNAs clarified from multivariable Cox regression analysis to predict 1‐, 3‐, and 5‐year survival rate of KIRC patients

We integrated risk scores from prognostic risk‐related ARlncRNAs signature and clinicopathological characteristics, included age, gender, grade, AJCC stage, and TNM stage. Subsequently, we performed univariate (Figure 4A) and multivariate Cox regression (Figure 4B), which showed that risk score, grade, and stage were independent prognostic factors (*p* < 0.05). Multiple ROC curves based on the risk score and clinicopathologic characteristics showed that the AUC value for prognostic risk‐related signature was 0.850, which was higher than the AUC value of gender (0.543), AJCC stage (0.678), T stage (0.631), N stage (0.607), and M stage (0.661) (Figure 4C). Furthermore, we found that patients with higher AJCC stages, grade, T stage, and M stage had higher risk scores than those with lower AJCC stages, grade, T stage, and M stage (*p* < 0.05). However, there were no significant differences in a different age, gender, and N stage (*p* > 0.05) (Figure 5). It also indicated that the prognostic risk‐related ARlncRNAs risk score model was connected to clinicopathological characteristics and could be independently used to predict prognostic outcomes in KIRC patients. Finally, we constructed two nomograms to predict 1‐, 3‐, and 5‐year survival times. One nomogram used risk score and seven prognostic‐related ARlncRNAs as variables and its C‐index value was 0.789 in training set and 0.724 in testing set (Figure 6A). The calibration plot showed good consistency between the actual observation and prediction by nomogram (Figure S1A‐C). The other nomogram used risk score and independent clinicopathological prognostic factors as variables and the C‐index value was 0.719 in training set and 0.715 in testing set, respectively (Figure 6b). The calibration curve also proved that the nomogram was reliable and accurate (Figure S1D‐F).

### 3.4 Construction of a LncRNA‐mRNA co‐expression network and functional enrichment analysis

**FIGURE 7:**
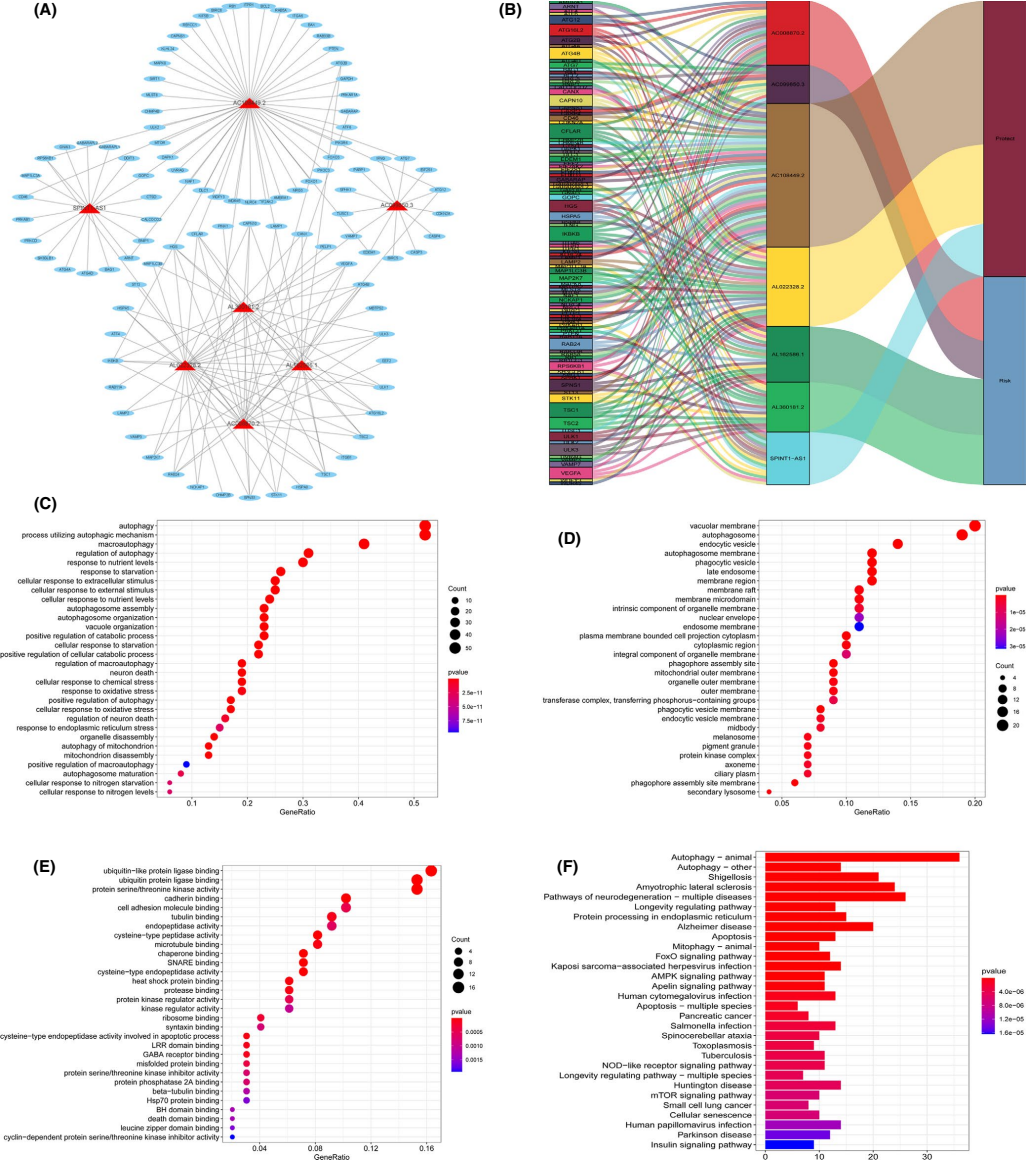
Construction of a LncRNA‐mRNA co‐expression network and functional enrichment analysis. (A) Diagrammatic plot displayed the lncRNA‐mRNA co‐expression network contained 165 lncRNA‐mRNA pairs formed by 7 prognostic risk‐related ARlncRNAs and 97 mRNAs. (B) Sankey diagram showed the relationship between 7 prognostic risk‐related ARlncRNAs, 97 mRNAs, and risk types (risk or protective). (C–E) Gene Ontology (GO) analysis of target mRNAs, which were co‐expressed with seven prognostic risk‐related ARlncRNAs, revealed the enriched (C) biological processes, (D) cell components, and (E) molecular functions. (F) Kyoto Encyclopedia of Genes and Genomes (KEGG) pathway analysis of target mRNAs, which were co‐expressed with seven prognostic‐related ARlncRNAs, revealed the enriched signaling pathways

We constructed a lncRNA‐mRNA co‐expression network contained 165 lncRNA‐mRNA pairs to investigate the potential biological function of seven prognostic risk‐related ARlncRNAs (Figure 7A). The Sankey diagram displayed the association between seven prognostic risk‐related ARlncRNAs and targeted mRNAs as well as risk types included risk or protective factors (Figure 7B).

GO enrichment analysis and KEGG pathway analysis of targeted mRNA were adopted and the top 30 terms were displayed. We found that the top five GO terms for biological processes were displayed in Figure 7C, included autophagy, a process utilizing autophagic mechanism, macro‐autophagy, autophagosome assembly, autophagosome organization. The top five GO terms for cellular components were autophagosome, autophagosome membrane, vacuolar membrane, phagophore assembly site, and phagocytic vesicle (Figure 7D). The top five GO terms for molecular functions were ubiquitin‐like protein ligase binding, ubiquitin‐protein ligase binding, protein serine/threonine kinase activity, chaperone binding, and snare binding (Figure 7E). According to KEGG analysis, the top five pathways included autophagy‐animal, autophagy‐other, shigellosis, amyotrophic lateral sclerosis, and pathways of neurodegeneration‐multiple diseases (Figure 7G).

### 3.5 Comparison of the predictive ability of the constructed predictive signature with the published prognostic models

**FIGURE 8:**
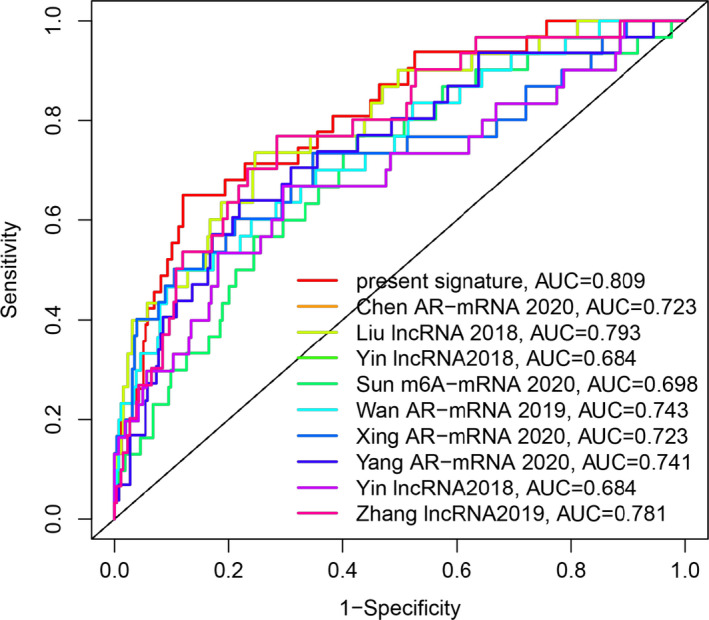
We compared the prognostic‐related ARlncRNAs signature with published predictive models in KIRC patients. The ROC curves showed that the present signature had higher prediction reliability and sensitivity than other published biomarkers

We compared the prognostic‐related ARlncRNAs signature with published predictive models in KIRC patients. The ROC curves showed that the present signature (AUC = 0.809) had higher predictive reliability and sensitivity than other published biomarkers (Chen et al^16^ autophagy‐related (AR) mRNA signature, AUC = 0.723; Liu, et al^17^ lncRNA signature, AUC = 0.723; Sun, et al^18^ immune‐related lncRNA, AUC = 0.709; Yin, et al^19^ lncRNA signature, AUC = 0.684; Sun, et al^20^ m6A‐related signature, AUC = 0.698; Wan, et al^21^ AR‐mRNA signature, AUC = 0.743;Xing, et al^22^ AR‐mRNA signature, AUC = 0.723; Yang, et al^23^ AR‐mRNA signature, AUC = 0.741; Zhang, et al^24^ lncRNA signature, AUC = 0.781; Figure 8).

## 4 CONCLUSIONS

Several studies have reported that autophagy played a controversial role in initiating and developing many tumors.^25^ Moreover, previous studies had explored the correlation between autophagy‐related genes and RCC.^26^ lncRNAs were involved in multiple biological processes, especially in tumors.^11^, ^27^ However, the correlation between the expression of ARlncRNAs and the prognosis status of patients with KIRC has not been established. In our present study, we identified seven prognostic‐related ARlncRNAs and constructed a prognostic risk score signature to predict the prognosis of the patients with KIRC accurately. We divided the samples into training group and testing group randomly and performed univariate Cox proportional hazards regression analysis in the training group, and we found that expressions of 146 lncRNAs were significantly linked with prognosis of KIRC patients. Then, we performed Lasso regression analysis and multivariate Cox proportional hazards regression analysis and identified seven prognostic‐related ARlncRNAs as independent prognostic factors for KIRC patients. Subsequently, we constructed the prognostic risk score model using the seven ARlncRNAs.

We validated the accuracy and reliability of the prognostic risk score signature using Kaplan–Meier survival curves and ROC curves in training set and testing set, respectively. The risk Score distribution and scatter dot plot showed the risk score and survival outcomes in high‐ and low‐risk score patients in training set and testing set to further validate the accuracy of patients with KIRC. Furthermore, we assessed the relationship between risk score from the prognostic signature and clinicopathological characteristics and found that risk score, grade, and AJCC stage were independent prognostic factors. Multiple ROC curves were performed and confirmed the prognostic reliability of risk score from prognostic risk‐related ARlncRNAs signature was superior to clinicopathological characteristics. Prognostic nomograms were constructed and used to predict the prognosis of patients with KIRC accurately. Simultaneously, we constructed a lncRNA‐mRNA co‐expression network to assess the correlation between prognostic‐related ARlncRNAs and targeted mRNA. Moreover, we performed GO and KEGG enrichment analysis to search for the main functions of the ARlncRNAs and downstream pathways. Collectively, we confirmed that the prognostic risk score signature could be a reliable and valuable diagnostic and therapeutic indicator for KIRC patients.

Among the seven prognostic risk‐related ARlncRNAs identified in the present study, AL162586.1, AL360181.2, AC008870.2, and AC099850.3 were risk factors upregulated in high‐risk score group, whereas AC108449.2, SPINT1‐AS1, and AL022328.2 were protective factors, which were downregulated in high‐risk score group. Besides, the expression of AL162586.1, AL360181.2, and AC008870.2 was positively correlated with KIRC patients’ survival (*p* < 0.05). These suggested that they might act as tumor suppressors in KIRC. lncRNA AC099850.3 has been reported as a member of the lncRNA‐miRNA‐mRNA competitive endogenous RNA network that might be useful in the diagnosis and treatment of squamous cell carcinoma of the tongue.^28^ Meanwhile, lncRNA AC099850.3 was a member of the ARlncRNAs prognostic model and could serve as an indicator of hepatocellular carcinoma diagnosis and treatment.^29^ The expression of SPINT1‐AS1 was negatively correlated with esophageal squamous cell carcinoma (ESCC) patients’ survival (*p* < 0.05), which suggested that they could act as tumor suppressors in ESCC.^30^ Furthermore, lncRNA AC108449.2 could be used as a member of the ARlncRNAs prognostic model and the immune‐related prognostic model for the diagnosis and treatment of bladder^31^ and kidney carcinoma,^18^ respectively. Additionally, Li et al. found that lncRNA SPINT1‐AS1 was downregulated in esophageal colorectal carcinoma tissues, suggesting that it might also be a marker of tumor prognosis,^32^ which was consistent with the result of this study. Besides, lncRNAs SPINT1‐AS1 was able to regulate Lapatinib sensitivity for pan‐cancer.^33^ No relevant studies have reported the prognostic role in the remaining three ARlncRNAs in tumors. Therefore, more studies were needed to elucidate exactly how these lncRNAs affect the prognostic outcomes of KIRC patients via autophagy. Moreover, after exploring published prognostic models of KIRC patients, we found that the present prognostic signature had high predictive accuracy, which could be a new prognostic marker for KIRC.

In conclusion, the strength of the present study was that we extracted data from public databases and used systematic statistical approaches to analyze the role of autophagy and lncRNAs in KIRC. The prognostic risk‐related signature based on seven ARlncRNAs was successfully constructed and accessed in testing set carefully. Prognostic nomograms were established and proved to be an effective and reliable clinical prediction tool, with which we could accurately predict the prognostic outcomes of patients with KIRC. In addition, the co‐expression network between ARlncRNAs and target mRNAs was constructed, and GO and KEGG functional enrichment analyses were performed, which provides a basis for future studies.

## CONFLICTS OF INTEREST

The authors report no conflict of interest.

## AUTHOR CONTRIBUTIONS

JunJie Yu was responsible for study design, data acquisition and analysis, and manuscript writing; JunJie Yu and WeiPu Mao performed bioinformatics and statistical analyses; JunJie Yu and WeiPu Mao prepared the figures and tables for the manuscript; Bin Xu and Chen Ming were responsible for the integrity of the entire study and manuscript review. All authors read and approved the final manuscript.

## Supporting information

Fig S1

## Data Availability

The data sets used and analyzed during the study are available from the corresponding author on reasonable request.
