# Supplementary material for: Construction and validation of an autophagy‐related long noncoding RNA signature for prognosis prediction in kidney renal clear cell carcinoma patients
Source: Cancer Med. 2021 Mar 2;10(7):2359–69. doi: 10.1002/cam4.3820 (PMC7982638; doi:10.1002/cam4.3820)
Supplement: Supplementary file 1 — Fig S1 [file CAM4-10-2359-s002.docx]

Figure S1


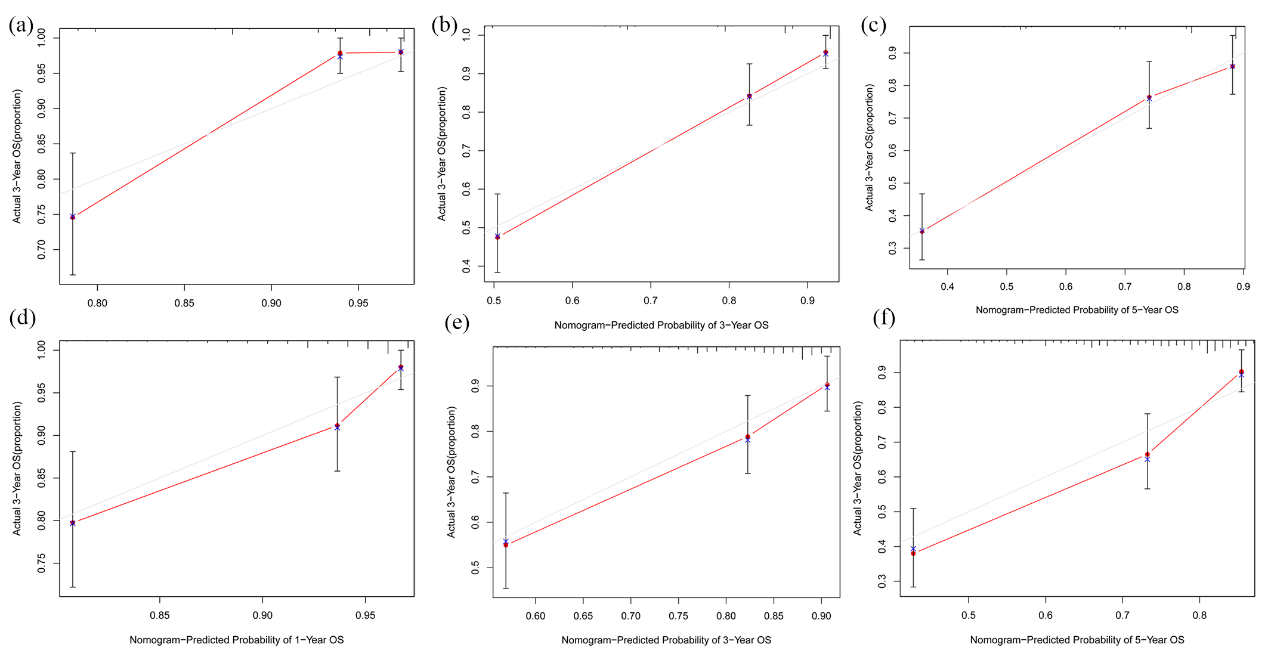


**Figure S1:** (a-c) The calibration curves of the nomogram constructed by figure5a displayed the concordance between predicted and observed 1-, 3- and 5-year survival outcomes. (d-e). The calibration curves of the nomogram constructed by figure5b displayed the concordance between predicted and observed 1-, 3- and 5-year survival outcomes.
