# Supplementary figures and images for: Construction and validation of an autophagy‐related long noncoding RNA signature for prognosis prediction in kidney renal clear cell carcinoma patients
Source: Cancer Med. 2021 Mar 2;10(7):2359–69. doi: 10.1002/cam4.3820 (PMC7982638; doi:10.1002/cam4.3820)

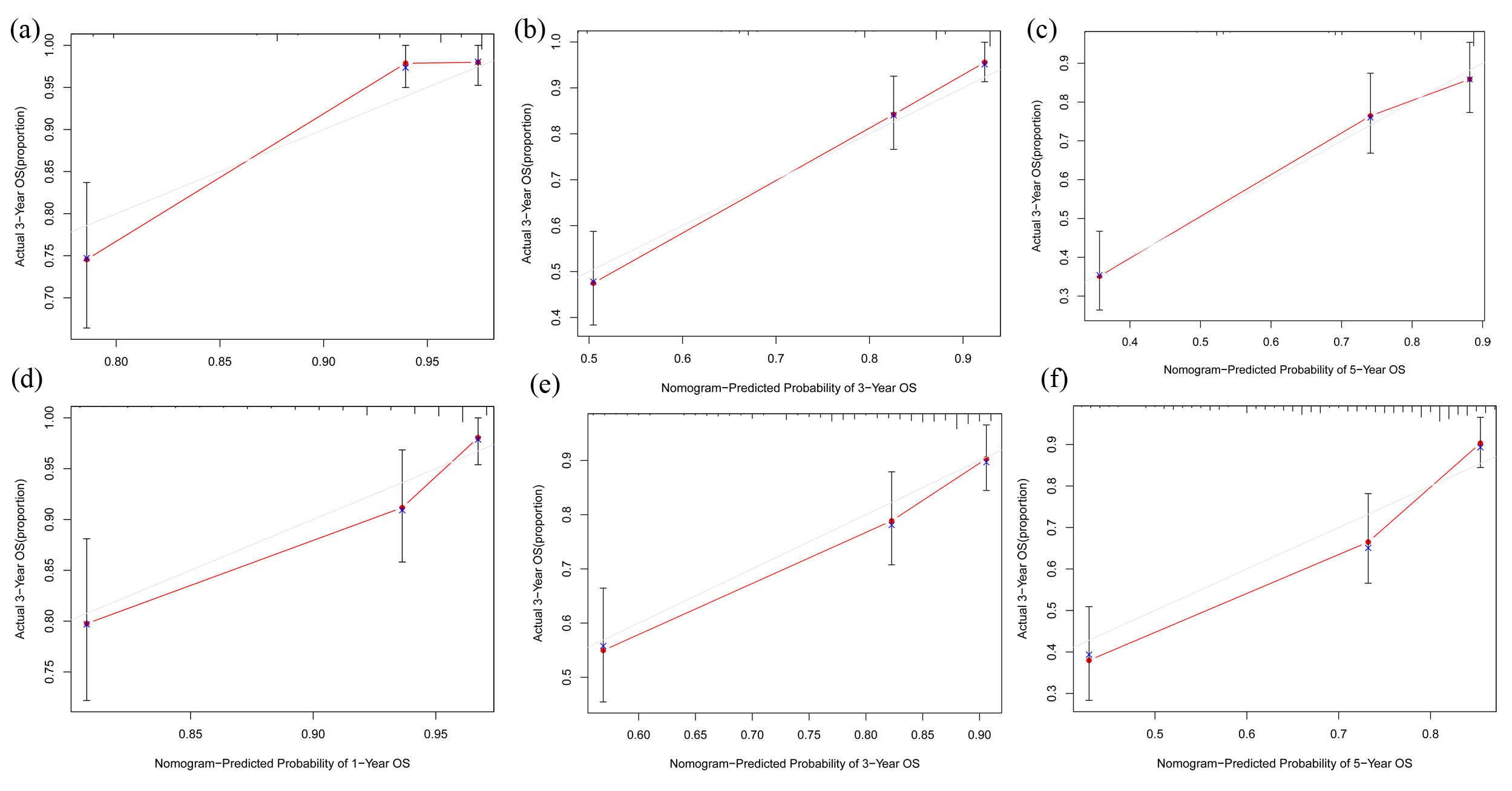

Supplement: Supplementary file 2 [file CAM4-10-2359-s001.tif]
